# The founder-cell transcriptome in the *Arabidopsis apetala1 cauliflower* inflorescence meristem

**DOI:** 10.1186/s12864-016-3189-x

**Published:** 2016-11-03

**Authors:** Anneke Frerichs, Rahere Thoma, Ali Taleb Abdallah, Peter Frommolt, Wolfgang Werr, John William Chandler

**Affiliations:** 1 Institute of Developmental Biology, University of Cologne, Cologne Biocenter, Zuelpicher Strasse 47b, D-50674 Cologne, Germany; 2 CECAD Research Center, University of Cologne, Joseph-Stelzmann-Str. 26, 50931 Cologne, Germany; 3 Present address: Department of Plant Breeding and Genetics, Max Planck Institute for Plant Breeding Research, Carl-von-Linné-Weg 10, D-50829 Cologne, Germany

**Keywords:** *apetala1 cauliflower*, *Bract*, *DORNRÖSCHEN-LIKE*, Fluorescence-activated cell sorting, Inflorescence meristem, Lateral organ founder cell, RNA-seq, Transcriptome

## Abstract

**Background:**

Although the pattern of lateral organ formation from apical meristems establishes species-specific plant architecture, the positional information that confers cell fate to cells as they transit to the meristem flanks where they differentiate, remains largely unknown. We have combined fluorescence-activated cell sorting and RNA-seq to characterise the cell-type-specific transcriptome at the earliest developmental time-point of lateral organ formation using *DORNRÖSCHEN-LIKE::GFP* to mark founder-cell populations at the periphery of the inflorescence meristem (IM) in *apetala1 cauliflower* double mutants, which overproliferate IMs.

**Results:**

Within the lateral organ founder-cell population at the inflorescence meristem, floral primordium identity genes are upregulated and stem-cell identity markers are downregulated. Additional differentially expressed transcripts are involved in polarity generation and boundary formation, and in epigenetic and post-translational changes. However, only subtle transcriptional reprogramming within the global auxin network was observed.

**Conclusions:**

The transcriptional network of differentially expressed genes supports the hypothesis that lateral organ founder-cell specification involves the creation of polarity from the centre to the periphery of the IM and the establishment of a boundary from surrounding cells, consistent with bract initiation. However, contrary to the established paradigm that sites of auxin response maxima pre-pattern lateral organ initiation in the IM, auxin response might play a minor role in the earliest stages of lateral floral initiation.

**Electronic supplementary material:**

The online version of this article (doi:10.1186/s12864-016-3189-x) contains supplementary material, which is available to authorized users.

## Background

The development of the aerial plant body depends on the activity of the shoot apical meristem (SAM), whereby pluripotent stem cells transit from the central stem-cell zone towards the periphery and become specified as lateral organ founder cells (LOFCs) depending on their precise position. Coordinated cell divisions within small groups of LOFCs create an organ primordium that then acquires fate [1]. In *Arabidopsis thaliana*, leaves are initiated during the vegetative phase and axillary meristems remain dormant; in contrast, the floral transition consists of biphasic meristem identity, in which secondary inflorescences initiate in the axils of cauline leaves in a pre-floral inflorescence phase and following the complete acquisition of reproductive competence, floral primordia are initiated in the axils of subtending bracts [2], whose outgrowth in *Arabidopsis* is subsequently suppressed. Thus, consistent with phytomer theory, the floral meristem (FM) can be considered as an axillary meristem, whose initiation depends on that of the cryptic bract [3]. Bract growth is known to be linked with floral organ initiation [4] and a genetic determinant of bract identity and growth, *LEAFY* (*LFY*), also regulates floral primordium formation.

Groups of LOFCs in the IM are characterised by transcription of the *DORNRÖSCHEN-LIKE* (*DRNL*) AP2-type transcription factor gene in a spiral phyllotaxy from near the centre of the IM towards the morphologically apparent stage 1 floral buttress [5]. Here, the population of *DRNL*-expressing LOFCs bifurcates into two foci; one at the tip of the floral buttress where the abaxial sepal will develop [6] and the other basally at the cryptic bract position. Bract development in *lfy* and *puchi* mutants disrupts the unidirectional sequence of first-whorl floral organ initiation of wild type [6], which suggests a complex developmental dynamism of founder-cell specification and overlapping positional information for the abaxial sepal and bract in the wild type IM. LOFC specification in the outer floral whorl of sepals occurs in the absence of stem-cell markers such as *CLAVATA3* (*CLV3*) or *WUSCHEL* (*WUS*) at the IM periphery, which regain activity after initation of the abaxial sepal, when a furrow separates the stage 2 primordium from the IM [7, 8].

A suitable genetic background in which to study the earliest stages of FM initiation is the *apetala1 cauliflower* (*ap1 cal*) double mutant, which overproliferates IMs before the delayed production of FMs [9]. The resulting inflorescence apices are massively enriched in synchronised IMs that specify LOFCs in a spiral phylotaxy at the IM periphery according to *DRNL* expression [6]. The *ap1 cal* genetic background has been combined with appropriate cell-type-specific fluorescent markers and used for fluorescence-activated cell sorting (FACS) coupled with microarray analysis to transcriptionally profile the meristem stem-cell niche [8, 10] or with chromatin immunoprecipitation analyses to identify the physical targets of MADS-box transcription factors [11]. The synchronisation of IMs in the *ap1 cal* apex restricts analyses to a short developmental window and the *DRNL::GFP-*expressing LOFCs can be separated via FACS from their non-expressing neighbours for comparative transcriptome analysis. This provides access to the earliest phase of cell-type specification in the IM peripheral zone.

The initiation of lateral organs involves the repression of the class I *KNOX* genes *SHOOTMERISTEMLESS* (*STM*) and *BREVIPEDICELLUS* (*BP*) by the ASYMMETRIC LEAVES1 (AS1) and AS2 transcription factors to promote cell differentiation [12]. In *Arabidopsis*, auxin is also a positional determinant, because polar auxin transport generates auxin response maxima at sites of incipient FM initiation [13] and mutation of the auxin polar transport and signalling components *PIN-FORMED1* (*PIN1*) and *MONOPTEROS* (*MP*) completely blocks the formation of FMs [14, 15]. The downstream signalling cascade from MP in lateral organ initiation is partially known and includes the LFY, AINTEGUMENTA (ANT), AINTEGUMENTA-LIKE6 (AIL6) and FILAMENTOUS FLOWER (FIL) transcription factors [16, 17]. However, auxin response is not the only phyllotactic signal, and it co-functions with cytokinin signalling via ARABIDOPSIS HISTIDINE PHOSPHOTRANSFER PROTEIN 6 (AHP6) [18]. *AHP6* is a target gene of DRNL [19] and the *AHP6* and *DRNL* expression domains only partially overlap with that of the *DR5* auxin response reporter and are more distal towards the IM periphery [6], indicating polarity with respect to auxin or cytokinin response. Polarity is an iterating scheme in lateral organ development in the IM, starting with progenitor cell divisions that occur near the central zone and defining an outward trajectory along an ad-/abaxial axis [20]. Furthermore, the polarity of the floral meristem is affected by several genes, including *BLADE ON PETIOLE1* (*BOP1*) and *BOP2* [21, 22], *YABBY* (*YAB*) [23] and *ETTIN* [24].

Despite the identification of some components of the gene regulatory networks (GRNs), including hormonal signals, which affect lateral organ initiation at the IM periphery, several problems remain: firstly, whether auxin or cytokinin signalling is causal or correlative with respect to LOFC specification; secondly, the relative timing of FM initiation in the axils of cryptic bracts within the IM, according to phytomer theory and thirdly, the basis of the interplay between founder-cell recruitment for the bract and FM, as is suggested by the altered series of sepal initiation in *puchi* and *lfy* mutants [6]. Resolving these issues is facilitated by detailed knowledge of the GRNs that are active in LOFCs compared to in the IM. Similar data are available at a single-cell resolution for specification of the hypophysis [25], lateral-root founder cells (reviewed in [26]), the endodermis/cortex initial [27] and the root phloem [28].

To optimise the resolution of studying the LOFC GRN, here, we have combined FACS using the *DRNL::GFP* founder-cell marker in the *ap1 cal* genetic background and RNA-seq, to capture the LOFC transcriptome at the earliest developmental time-point of lateral organ formation at the IM periphery. Next-generation sequencing, and especially RNA-seq [29], has become the method of choice for genome-wide transcriptional profiling, due to its ability to quantitate transcript expression over a large dynamic expression range and has to date been used in *Arabidopsis* to characterise the transcriptomes of pollen [30] and wild-type or homeotic mutant flowers [31]. We show here, that the combined use of FACS/RNA-seq is suitable to address cellular decisions in the SAM at a resolution not previously achieved. The LOFC transcriptome data represent a unique resource that allows the interrogation of aspects of transcriptional control and the molecular pathways that enable founder-cell specification, and that in comparison to the *ap1 cal* IM transcriptome, depicts the molecular repertoire that accompanies the cellular specification of bract, sepal or FM tissue at the IM periphery.

## Results

### Isolation of *DRNL::GFP*-expressing cells from *ap1 cal* inflorescence apices

**Fig. 1:**
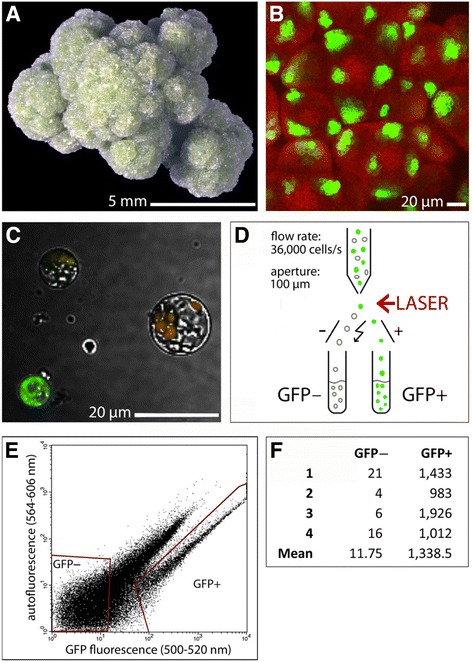
**a** An *ap1 cal* inflorescence at the stage used for following fluorescence-activated cell sorting (FACS), illustrating the massive overproliferation of inflorescence meristems before the initiation of floral meristems. **b** A confocal image of *DRNL::erGFP* expression in the *ap1 cal* inflorescence showing *DRNL* expression in phyllotactic founder-cell populations of incipient lateral organs in reiterating inflorescence meristems; red represents chlorophyll autofluorescence. **c** A confocal image of GFP+ and GFP– protoplasts following FACS of protoplasts from *ap1 cal*/*DRNL::GFP* inflorescences. **d** A schematic work flow to show the separation of GFP+ and GFP– cells via FACS. **e** A FACS scattergraph of the protoplasts showing the fractions collected for RNA-seq according to the output from GFP fluorescence and autofluorescence. **f** A table showing the absolute counts of *GFP* sequence reads in the RNA-seq data of GFP+ and GFP– protoplasts following FACS from four independent samples

The cauliflower inflorescence phenotype of the *ap1 cal* double mutant (Fig. 1a) results from the initiation of lateral meristems at the IM periphery that retain IM identity and initiate secondary or tertiary IM meristems in a spiral phyllotaxy, which is revealed by imaging of the lateral organ founder-cell (LOFC) marker *DRNL::GFP* (Fig. 1b). Expression of *DRNL::GFP* continues for 1–2 h after protoplasting (Fig. 1c) which allows the GFP+ protoplasts to be collected via FACS, as schematically depicted in Fig. 1d. A representative scattergraph shows the separation of GFP+ and GFP− protoplasts in Fig. 1e and based on several analytical and preparative FACS experiments, GFP+ protoplasts represent maximally 10 % of the total protoplasts. The mean total RNA yield in four preparative FACS experiments from GFP+ and GFP– cells was 278.4 ng/100,000 protoplasts and individual samples were normalised prior to cDNA synthesis and RNA-seq analyses. The data discussed below are based on at least 65 million mapped 100-bp reads for each RNA sample in four biological replicates. As confirmation of the FACS efficiency (Fig. 1f), the mean number of absolute GFP sequence reads increased more than 100-fold from ~12 in GFP− protoplasts to ~1,338 reads in GFP+ protoplasts.

**Fig. 2:**
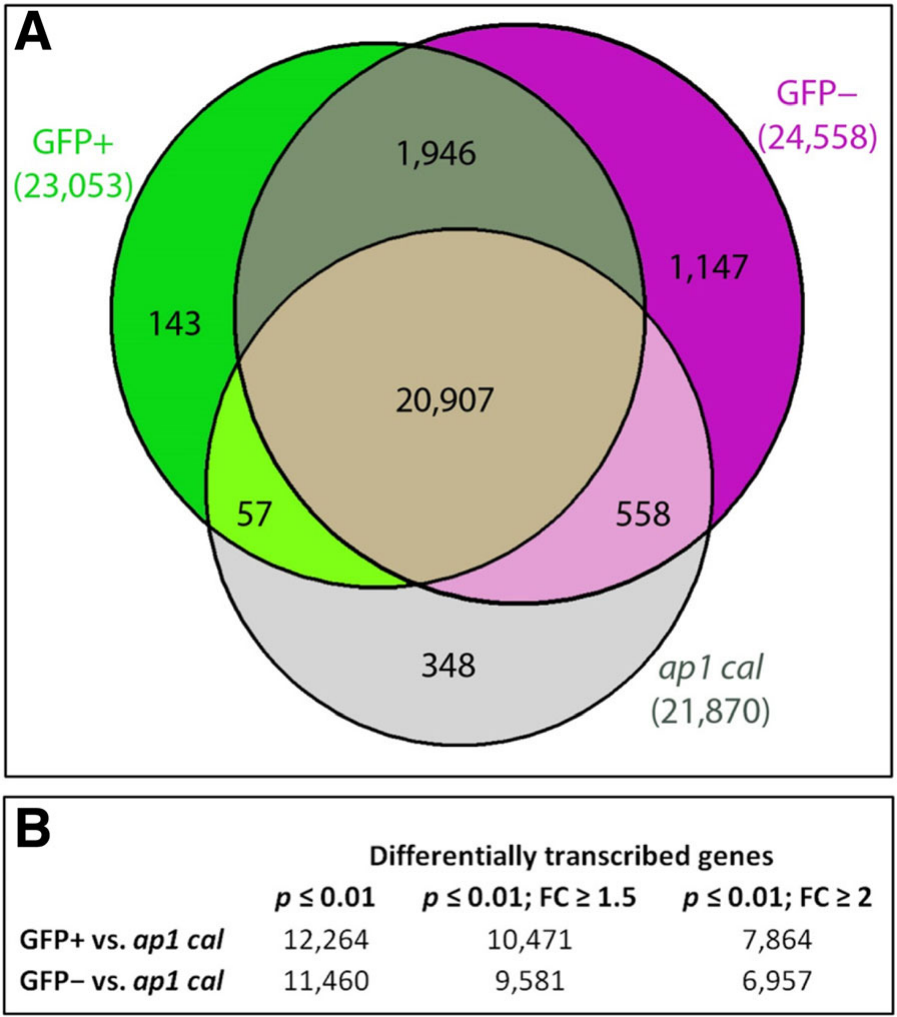
**a** A Venn-diagram depicting the overlap in the number of transcribed genes (normalised read counts ≥ 1) in populations of GFP+ protoplasts (23,053 transcribed genes in total), GFP– protoplasts (24,558 in total) and unsorted *ap1 cal* inflorescence apices (21,870 in total). **b** Summary of the number of differentially transcribed genes at different probability and cut-off values (*p* ≤ 0.01; *p* ≤ 0.01and FC ≥ 1.5, or *p* ≤ 0.01 and FC ≥ 2.0) when the GFP+ or GFP– transcriptome was compared with that of the unsorted *ap1 cal* IM

To estimate the consequences of protoplast preparation on the transcriptome, we compared the RNA-seq data from GFP+ and GFP– protoplasts to those from RNA directly obtained from unsorted whole apices of *ap1 cal* inflorescences and focussed on nuclear genes, i.e., we excluded 133 chloroplast and 146 mitochondrial genes from comparative analyses. Out of 33,279 nuclear *Arabidopsis* genes, 21,870 were transcribed in unsorted *ap1 cal* inflorescence apices (Fig. 2a) and the number of expressed genes increased to 23,053 in GFP+ protoplasts, or 24,558 in GFP– protoplasts, calculated as normalised read counts (NRC; see Methods ≥1).

**Fig. 3:**
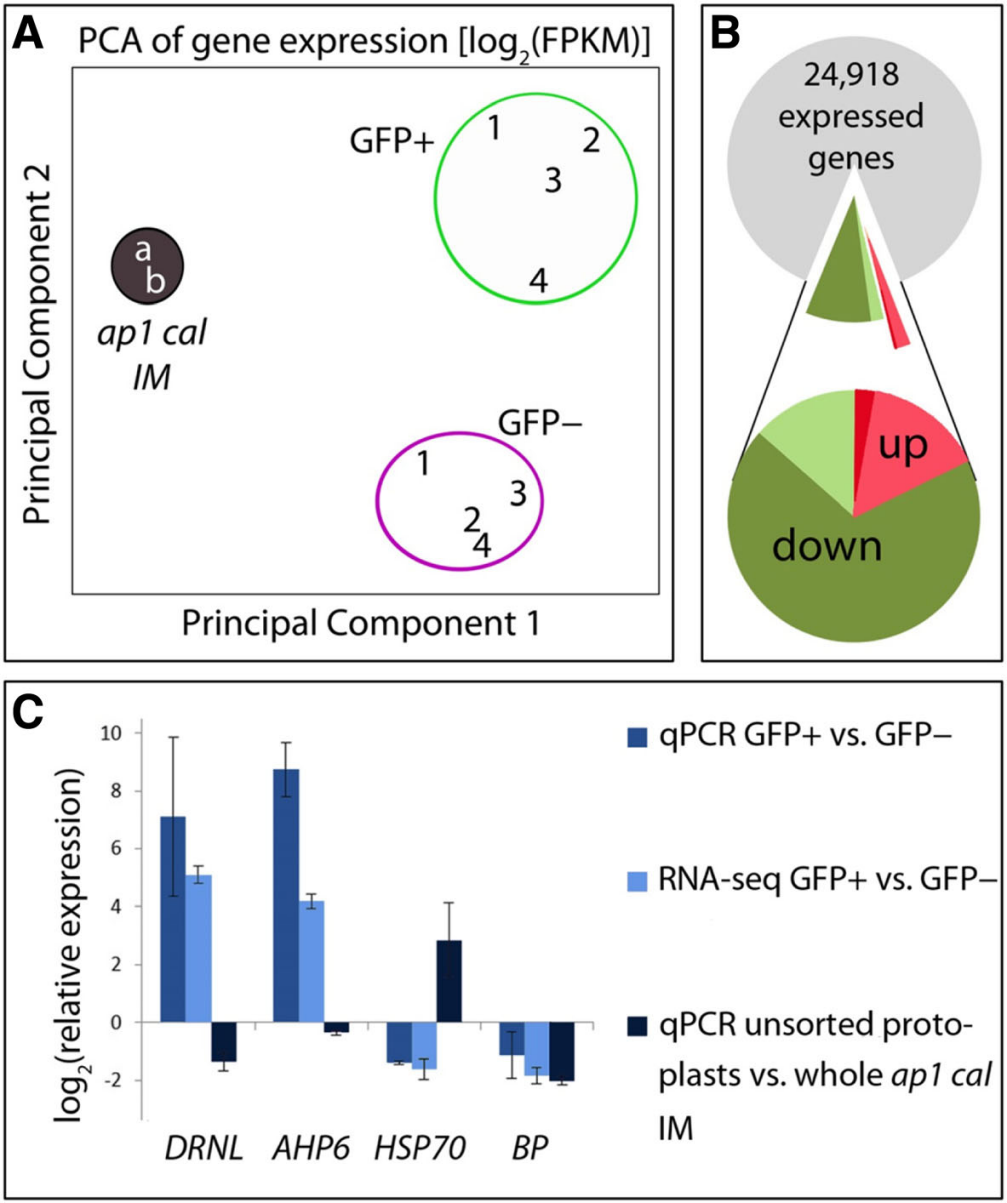
**a** Principal component analysis (PCA) of global gene expression from different biological replicates of RNA samples for RNA-seq. Expression estimates were log-transformed and subjected to PCA using a covariance matrix. The biological replicates clustered together, whereas the different cell types were distinct. **b** A pie chart representing the number of differentially expressed genes from *DRNL::GFP ap1 cal* apices. Out of 24,918 expressed transcripts (NRC ≥ 1 in the GFP+/GFP− comparison in at least one of the type of protoplasts), the proportion significantly up-regulated (*p* ≤ 0.01; fold-change ≥ 1.5 light and dark-red; fold-change ≥ 2.0 dark red) or downregulated (*p* ≤ 0.01; fold-change ≥ 1.5 light and dark-green; fold-change ≥ 2.0 dark green) in GFP+ protoplasts compared to GFP– protoplasts is shown. **c** The log_2_ (relative transcript expression) for *DÖRNRÖSCHEN-LIKE* (*DRNL*), *ARABIDOPSIS HISTIDINE PHOSPHOTRANSFER PROTEIN 6* (*AHP6*), *HEAT-SHOCK PROTEIN70* (*HSP70*) and *BREVIPEDICELLUS* (*BP*) is depicted as a ratio from GFP+/GFP− protoplasts determined by qPCR or taken from the RNA-seq data. Additionally, differences in the expression of the four genes in unsorted protoplasts vs. whole apices of *ap1 cal* inflorescences were analysed via qPCR

In bilateral comparisons between the *ap1 cal* transcriptome and those of GFP+ and GFP– protoplasts, a total of 20,907 genes were commonly transcribed. Cell-wall digestion for protoplast preparation thus increased the number of transcribed genes (NRC ≥ 1) by 2,146 to 23,053 in GFP+ protoplasts and by 3,651, to 24,558 genes in GFP− protoplasts relative to unsorted *ap1 cal* IMs. The majority of the genes activated by protoplasting (1,946 in Fig. 2a), were shared by both GFP+ and GFP− protoplast populations and according to gene ontology (GO) enrichment analysis for the domain “Cellular component”, mainly group into the functional categories: *nucleus*, *other membranes*, *other cytoplasmic components* and *extracellular*. The differentially expressed transcripts activated by protoplasting in GFP+ and GFP– protoplasts cannot be distinguished by specific GO categories and possibly relate to a higher cell-type diversity in the GFP− sample. Protoplasting not only activated additional genes, but also affected differential gene expression; at a threshold of *p* ≤ 0.01, 12,264 nuclear genes were differentially transcribed in the GFP+/*ap1 cal* comparison and slightly fewer genes (11,460) in the GFP−/*ap1 cal* comparison. Implementing a minimal fold-change (FC) ≥ 1.5 (*p* ≤ 0.01), reduced the number of genes responding to cell wall digestion to 10,471 and 9,581 genes in the GFP+/*ap1 cal* and GFP−/*ap1 cal* comparisons*,* or at a higher stringency of FC ≥ 2 (*p* ≤ 0.01), to 7,864 or 6,957 differentially expressed genes, respectively (Fig. 2b). In each case, more common genes were present in the transcriptome of GFP− protoplasts and the unsorted *ap1 cal* IM than between GFP+ protoplasts and the *ap1 cal* IM, which possibly relates to the small fraction of cells expressing *DRNL::GFP* in the *ap1 cal* IM (Fig. 1b), depicted in the FACS scattergraph (Fig. 1e). A principal components analysis (Fig. 3a) on gene expression showed that the biological replicates for GFP+ and GFP− protoplasts and for *ap1 cal* apices clustered together, but that each set of cell-specific samples was distinct, demonstrating the reproducibility and statistical reliability of the data. The complete RNA-seq dataset is available at Gene Expression Omnibus (http://www.ncbi.nlm.nih.gov/geo/).

### Digital transcriptional differences between *DRNL::GFP*-positive and -negative cells

A direct comparison of transcripts in *DRNL::GFP*+ and *DRNL::GFP*− protoplasts at a stringency of FC ≥ 2.0 (*p* ≤ 0.01) revealed 109 activated and 2,801 repressed genes in GFP+ cells, out of a total of 24,918 expressed genes (NRC ≥ 1), depicted by dark red and dark green sectors, respectively, in Fig. 3b. The highest enrichment was observed for *DRNL* (FC = +34.32), whose expression increased from 4.68 NRC in *DRNL::GFP*– to 199.5 NRC in *DRNL::GFP*+ protoplasts, showing that transcription of the endogenous *DRNL* gene reflects expression of the *DRNL::GFP* marker. The next-highest differentially expressed gene was *AHP6* (FC = +18.08), which is a DRNL target that is transcribed in a similar pattern to *DRNL* in the IM periphery. The upregulated genes were enriched for transcription factors and included *SHORT VEGETATIVE PHASE* (*SVP*) (FC = +2.16), associated with meristem identity, whereas *LEAFY* (*LFY*) (FC = +1.98) remained below the FC ≥ 2.0 threshold and similarly, auxin response factors (ARFs) showed no significant changes above a FC ≥ 1.5 (*p* ≤ 0.01). We therefore considered an FC = 2.0 to be too stringent and to exclude relevant differentially expressed transcripts and we lowered the threshold to FC ≥ 1.5 (*p* ≤ 0.01), which increased the fraction of up-regulated genes over six-fold, from 110 to 718, whereas the number of downregulated genes only increased by 20 %, from 2,801 to 3,356 (Fig. 3b). To validate the RNA-seq data, we selected a sub-set of 18 genes, including 13 upregulated and five downregulated transcripts and analysed their expression by qRT-PCR [see Additional file 1]. Despite quantitative differences, qRT-PCR data confirmed the up- or downregulation determined by RNA-seq. The comparative RNA-seq and qRT-PCR data for *DRNL* and *AHP6*, the most upregulated genes in GFP+ protoplasts, and the meristem marker *BP* that is downregulated in GFP+ protoplasts, are shown in Fig. 3c. As a general stress-responsive marker, we included the gene encoding *HEAT-SHOCK PROTEN 70* (*HSP70*), which is downregulated in GFP+ protoplasts, but is highly upregulated (FC = +6.27) following cell-wall digestion.

**Fig. 4:**
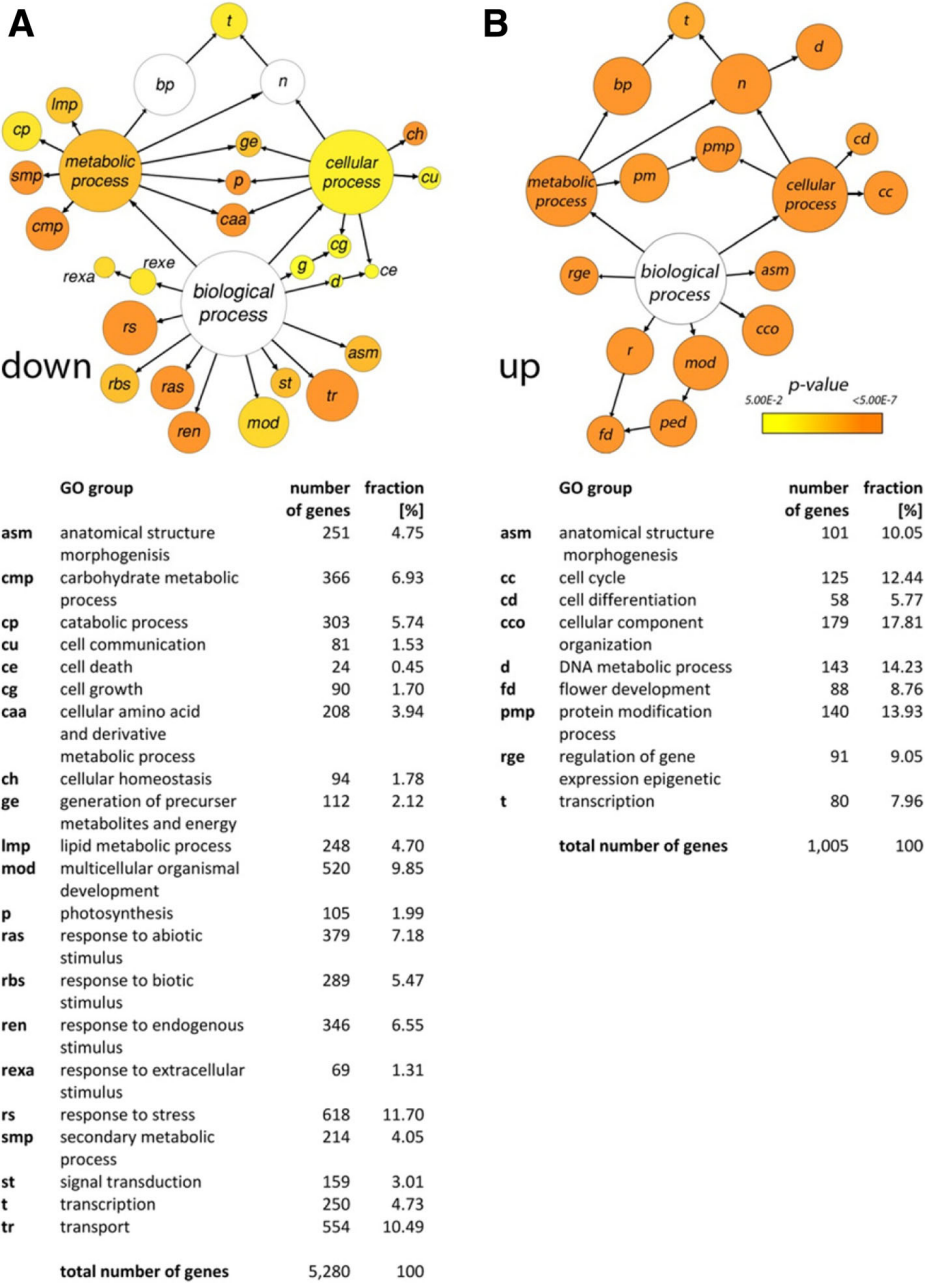
Networks based on enriched gene ontology (GO) categories of genes in *DRNL::GFP*-expressing cells compared to non-*DRNL::GFP*-expressing cells with a fold-change greater than 1.5 (*p* ≤ 0.01). **a** downregulated genes; **b** up-regulated genes. The GO terms were identified using BiNGO and visualised using Cytoscape. The circle diameter is proportional to the number of transcripts in each GO category according to TAIR10 annotation and the colour represents the *p*-value of enrichment. The number and fraction of genes in each category are summarised below the diagram

Gene ontology (GO) enrichment analysis for the GO-domain “Biological process” was compared for down- (Fig. 4a) and upregulated (Fig. 4b) genes (FC ≥ 1.5, *p* ≤ 0.01). We used the Biological Networks Gene Ontology tool (BiNGO) [32] to assess the over-representation of GO categories in subgraphs of biological networks; BiNGO depicts the fraction of genes in each GO category by circle size and the circle colour indicates the statistical significance. The resulting network or GO distribution clearly differs between the 716 up- and 3,356 downregulated genes; only two GO categories are shared by both groups: *anatomical structure morphogenesis* and *transcription*, the latter containing 4.73 % of down- and 7.96 % of upregulated genes. Considering only terminal nodes, the over-representation of genes in the GO categories *cell differentiation, flower development*, *cell component organisation*, *cell cycle* and *DNA metabolic process* suggest that the upregulated genes in *DRNL::GFP-*expressing cells favour primordium or floral differentiation, anisotropic growth or cell cycle progression/division for morphogenesis.

In contrast, the over-represented GO categories in the downregulated gene group are: *response to stress, response to biotic, abiotic, endogenous* and *extra cellular stimuli*, or *signal transduction*. The concerted reduction in transcript numbers in these GO categories in GFP+ cells suggests that they respond differentially to positional information than their surrounding meristematic neighbours that do not express *DRNL*. Furthermore, the downregulation of genes in the GO categories *transport, lipid*, *secondary* or *carbohydrate metabolic processes* and *catabolic process* suggests that *DRNL-*expressing cells also differ physiologically and metabolically relative to meristematic cells in the *ap1 cal* IM. These GO preferences support the interpretation that during the acquisition of functional autonomy from the meristem, *DRNL-*expressing cells acquire different signalling networks and show altered cellular physiology and metabolism.

### Differential gene expression supports the lateral organ founder-cell identity of *DRNL::GFP*-positive protoplasts

Many up-regulated genes in GFP+ protoplasts encode transcription factors and provide insight into correlative changes in regulatory networks that accompany *DRNL* activation (FC = +34.32) at the IM periphery (Table 1). In addition to the dramatic transcriptional upregulation of the DRNL target gene *AHP6* (FC = +18.08), many of the differentially expressed transcripts have functions in meristem identity and function, or in establishing polarity or boundaries (Table 1).

**Table 1:** Differentially regulated transcripts in *DRNL::GFP*-positive protoplasts (fold change ≥ 1.5; *p* ≤ 0.01) compared to *DRNL::GFP*− protoplasts that have functions associated with meristem maintenance and identity, polarity, boundary formation, hormones, vasculature, epigenetic changes and that generate miRNAs

Gene	Alias	Locus	Fold change
Meristem maintenance and identity/floral markers			
*ASYMMETRIC LEAVES2*	***AS2***	***At1g65620***	**+2.55**
*FLOWERING PROMOTING FACTOR1*	*FPF1*	*At5g24860*	+2.38
*SHORT VEGETATIVE PHASE*	***SVP***	***At2g22540***	**+2.16**
*REPRODUCTIVE MERISTEM1*	*REM1*	*At3g19184*	+2.10
*AINTEGMUMENTA*	***ANT***	***At4g37750***	**+2.08**
*LEAFY*	***LFY***	***At5g61850***	**+1.98**
*AINTEGUMENTA-LIKE6*	***AIL6***	***At5g10510***	**+1.96**
*PISTILLATA*	***PI***	***At5g20240***	**+1.93**
*REGULATOR OF AXILLARY MERISTEMS1*/*MYB37*	***RAX1***	***At5g23000***	**+1.86**
*BASIC PENTACYSTEINE 3*	***BPC3***	***At1g68120***	**+1.85**
*UNUSUAL FLORAL ORGANS*	***UFO***	***At1g30950***	**+1.85**
*BRCA1-ASSOCIATED RING DOMAIN1*	*BARD1*	*At1g04020*	+1.83
*CAULIFLOWER*	***CAL***	***At1g26310***	**+1.82**
*REPRODUCTIVE MERISTEM3*	*REM3*	*At5g58280*	+1.82
*LATERAL MERISTEM IDENTITY1*	***LMI1***	***At5g03790***	**+1.79**
*ULTRAPETALA1*	*ULT1*	*At4g28190*	+1.77
*APETALA1*	***AP1***	***At1g69120***	**+1.60**
*LATERAL MERISTEM IDENTITY2*	***LMI2***	***At3g61250***	***+*** **1.55**
*TUBBY8*	*TUB8*	*At1g16070*	+1.52
*SHOOTMERISTEMLESS*	***STM***	***At1g62360***	**−1.54**
*BARELY ANY MERISTEM3*	*BAM3*	*At4g20270*	−2.89
*BREVIPEDICELLUS*/*KNAT1*	***BP***	*At4g08150*	**−3.57**
Polarity			
*YABBY5*	***YAB5***	***At2g26580***	**+14.01**
*NUBBIN*	*NUB*	*At1g13400*	+5.81
*BLADE-ON-PETIOLE2*	***BOP2***	***At2g41370***	***+*** **5.44**
*BLADE-ON-PETIOLE1*	***BOP1***	***At3g57130***	***+*** **5.19**
*FILAMENTOUS FLOWER* (*YABBY1*)	***FIL***	***At2g45190***	**+4.37**
*JAGGED*	***JAG***	***At1g68480***	***+*** **3.32**
*PUCHI*	***PUCHI***	***At5g18560***	***+*** **3.25**
*KNOX ARABIDOPSIS THALIANA MEINOX*	*KNATM*	*At1g14760*	+3.17
*HOMEOBOX GENE1*	***ATH1***	***At4g32980***	**+2.90**
*PRESSED FLOWER*	*PRS*	*At2g28610*	+2.40
*YABBY3*	***YAB3***	***At4g00180***	**+2.14**
*ETTIN*	***ETT***	***At2g33860***	**+1.87**
*KANADI3*	*KAN3*	*At4g17695*	−3.52
Boundary genes			
*SUPERMAN*	***SUP***	*At3g23130*	**+3.07**
*JAGGED LATERAL ORGANS* (*LBD30*)	***JLO***	***At4g00220***	**+2.38**
*LATERAL BOUNDARY DOMAIN18*	*LBD18*	*At2g45420*	+2.21
*GROWTH REGULATING FACTOR2*	*GRF2*	*At4g37740*	*+*1.95
*GROWTH REGULATING FACTOR5*	*GRF5*	*At3g13960*	+1.86
*LATERAL ORGAN JUNCTION*	*LOJ*	*At2g39230*	+1.68
*GROWTH REGULATING FACTOR1*	*GRF1*	*At2g22840*	+1.63
*CUP-SHAPED COTYLEDON1*	***CUC1***	***At3g15170***	**+1.54**
*PETAL LOSS*	***PTL***	***At5g03680***	**−1.63**
*LATERAL ORGAN BOUNDARY*	***LOB***	***At5g63090***	**−8.87**
Auxin			
*MONOPTEROS*	***MP***	***At1g19850***	**+1.67**
*IAA CARBOXYLMETHYLTRANSFERASE1*	*IAMT 1*	*At5g55250*	−4.64
Brassinosteroids			
*BRI1-LIKE1*	*BRL1*	*At1g55610*	*+1.59*
*BR ENHANCED EXPRESSION2*	*BEE2*	*At4g36540*	*−3.97*
*BR ENHANCED EXPRESSION1*	*BEE1*	*At1g18400*	−8.15
Cytokinins			
*HISTIDINE PHOSPHOTRANSFER PROTEIN6*	***AHP6***	***At1g80100***	**+18.08**
*ISOPENTENYLTRANSFERASE7*	***IPT7***	***At3g23630***	**−7.25**
*ISOPENTENYLTRANSFERASE3*	*IPT3*	*At3g63110*	−13.47
Gibberellins			
*GIBBERELLIN OXIDASE3*	*GA3ox3*	*At4g21690*	+4.13
*GIBBERELLIN 2-OXIDASE4*	*GA2ox4*	*At1g47990*	+3.24
*GIBBERELLIN 20-OXIDASE2*	*GA20ox2*	*At5g51810*	+2.16
*GIBBERELLIN 3-OXIDASE1*	*GA3ox1*	*At1g15550*	+1.97
*GIBBERELLIN 2-OXIDASE2*	*GA2ox2*	*At1g30040*	+1.58
Other transcription factors			
*SHI-RELATED SEQUENCE4*	*SRS4*	*At2g18120*	+3.61
*STYLISH1*	***STY1***	***At3g51060***	**+2.74**
*SHI-RELATED SEQUENCE7*	*SRS7*	*At1g19790*	+2.21
*INFLORESCENCE DEFICIENT IN ABSCISSION* (*IDA*)-*LIKE 2*	*IDL2*	*At5g64667*	+1.93
*SHORT INTERNODES*	*SHI*	*At5g66350*	+1.92
MiRNAs and the siRNA precursor *TAS3A*			
*TAS3A*		*At3g17185*	−2.21
*MiR390A*		*At2g38325*	−2.73
*MiR159*/*159B*		*At1g18075*	−2.86
*MiR172D*		*At3g55512*	−3.06
*MiR156E*		*At5g11977*	−3.20
*MiR172*/*172B*		*At5g04275*	−5.55
*MiR164*/*164B*		*At5g01747*	−5.88
*MiR319*/*319B*		*At5g41663*	−6.29
*MiR160*/*160C*		*At5g46845*	−8.44
*MiR156C*		*At4g31877*	−11.49
*MiR172*/*172A*		*At2g28056*	−21.01
Vascular development			
*EPIDERMALPATTERNING LIKE FACTOR*-*LIKE 6*	*EPFL6*	*At2g30370*	−2.26
*REDUCED IN LATERAL GROWTH1*	*RUL1*	*At5g05160*	−2.44
*TDIF-RECEPTOR/PHLOEM INTERCALATED WITH XYLEM*	*TDR/PXY*	*At5g61480*	−2.81
*CLAVATA3/EMBRYO SURROUNDING REGION41*	*CLE41*	*At3g24770*	−2.84
*XYLEM CYSTEINE PEPTIDASE1*	*XCP1*	*At4g35350*	−3.02
*CLAVATA3/EMBRYO SURROUNDING REGION44*	*CLE44*	*At4g13195*	−3.24
*HIGH CAMBIAL ACTIVITY2*	*HCA2*	*At5g62940*	−6.05
*NAC SECONDARY WALL THICKENING PROMOTING FACTOR1*	*NST1*	*At2g46770*	−6.45
*VASCULAR RELATED NAC DOMAIN6*	*VND6*	*At5g62380*	−7.53
*VASCULAR RELATED NAC DOMAIN7*	*VND7*	*At1g71930*	−7.76
*XYLEM CYSTEINE PEPTIDASE2*	*XCP2*	*At1g20850*	−7.86
*WUSCHEL-RELATED HOMEOBOX4*	*WOX4*	*At1g46480*	−23.91
Epigenetic regulation			
*DECREASED DNA METHYLATION1*	*DDM1*	*At5g66750*	+1.91
*SET DOMAIN GROUP4*	*SDG4*	*At4g30860*	+1.82
*VARIANT IN METHYLATION2*	*VIM2*	*At1g66050*	+1.78
*VARIANT IN METHYLATION6*	*VIM6*	*At4g08590*	*+*1.77
*VARIANT IN METHYLATION1*	*VIM1*	*At1g57820*	*+*1.75
*VARIANT IN METHYLATION3*	*VIM3*	*At5g39550*	*+*1.73
*DNA METHYLTRANSFERASE1*	*MET1*	*At5g49160*	+1.71
*CHROMOMETHYLASE3*	*CMT3*	*At1g69770*	*+*1.63
*KRYPTONITE*	*KYP*	*At5g13960*	+1.53
*SET DOMAIN PROTEIN35*	*SDG35*	*At1g26760*	+1.52
*DEMETER-LIKE PROTEIN3*	*DML3*	*At4g34060*	−4.58

**Fig. 5:**
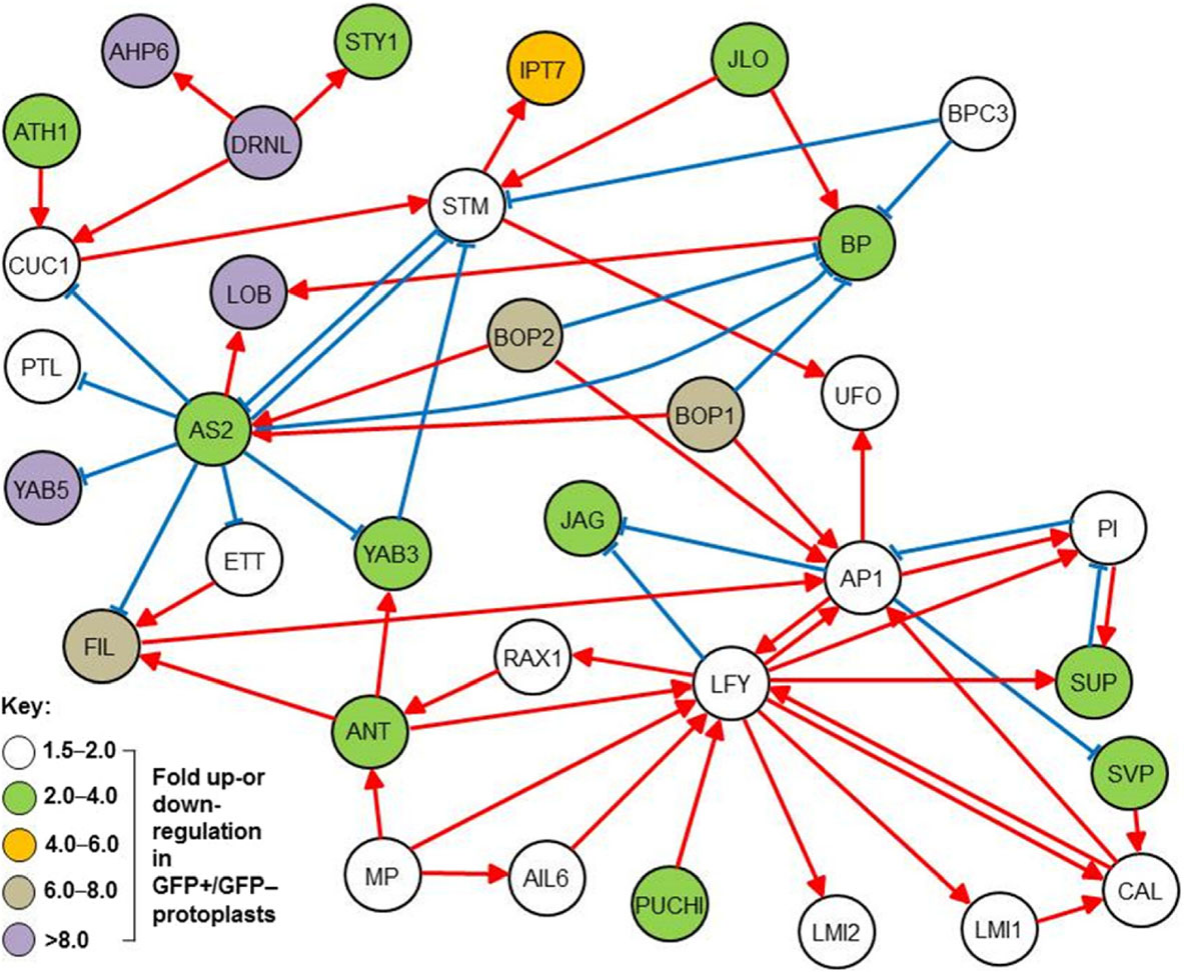
The gene network within *DRNL::GFP*-positive founder cells in the *ap1 cal* IM. Thirty-four significantly up- or downregulated DEGs were used to construct a high-confidence transcriptional network based on the Arabidopsis Transcriptional Regulatory Map (ATRM; Jin et al., [33]). The degree of up- or downregulation is depicted according to colour. Red arrows represent a positive regulation and blue bars a negative regulation of the target gene. Gene abbreviations are the same as in Table 1

Loci in bold represent genes used for the network in Fig. 5

The floral meristem identity genes *LEAFY* (*LFY*), or *APETALA1* (*AP1*) and *CAULIFLOWER* (*CAL*), which are inactive in the *ap1 cal* mutant background due to EMS mutations in the protein coding region were upregulated in GFP+ cells. Similarly, *UNUSUAL FLORAL ORGANS* (*UFO*), *LATERAL MERISTEM IDENTITY1* (*LMI1*), *LMI2*, *REPRODUCTIVE MERISTEM1* (*REM1*), *REM3*, *BRCA1-ASSOCIATED RING DOMAIN1* (*BARD1*), which restricts *WUS* expression, *BASIC PENTACYSTEINE3* (*BPC3*), *AINTEGUMENTA-LIKE6* (*AIL6*) and the axillary meristem marker *REGULATOR OF AXILLARY MERISTEMS1*/*MYB37,* were also upregulated. The reduced transcript levels of the two *KNOX* meristematic marker genes *BREVIPEDICELLUS* (*BP*) and *SHOOTMERISTEMLESS* (*STM*) and *BARELY ANY MERISTEM3* (*BAM3*), associated with meristematic stem cells, suggests that an increase in *DRNL::GFP* activity coincides with the loss of meristem identity.

Most of the polarity determinants were upregulated in *DRNL*-expressing cells (Table 1), including: *YABBY* (*YAB*) 5, *YAB3*, *FILAMENTOUS FLOWER* (*FIL*/*YAB1*), *BLADE-ON-PETIOLE1* (*BOP1*), *BOP2*, *AINTEGUMENTA* (*ANT*), *PUCHI*, *JAGGED* (*JAG*), the homeodomain-less *KNOX* gene *KNATM*, *PRESSED FLOWER* (*PRS/WOX3*), *HOMEOBOX GENE1* (*ATH1*) and the auxin response factor *ETTIN* (*ETT*). Among the polarity genes, *KANADI3* (*KAN3*) was significantly downregulated, as were the boundary genes *PETAL LOSS* (*PTL*) and *LATERAL ORGAN BOUNDARY* (*LOB*).

Similarly, the expression of genes associated with organ boundaries was significantly correlated with *DRNL* activation at the IM periphery, i.e., *JAGGED LATERAL ORGANS* (*JLO = LBD30*), *LATERAL BOUNDARY DOMAIN18* (*LBD18*) and *SUPERMAN* (*SUP*), *LATERAL ORGAN JUNCTIONS* (*LOJ*), *CUP-SHAPED COTYLEDON1* (*CUC1*), *GROWTH REGULATING FACTOR1* (*GRF1*), *GRF2* and *GRF5.*

The expression of floral organ identity genes such as *SEPALLATA* (*SEP*) *1*–*4*, *AP2*, *AP3* and *AGAMOUS* was not significantly altered according to the criteria of FC ≥ 1.5; *p* ≤ 0.01, as a further confirmation that *ap1 cal* IMs were harvested at an early morphological stage preceding FM and floral organ initiation. In summary, the transcriptional differences in characterised functional markers observed between GFP+ and GFP− cells separated by FACS, confirm that *DRNL*-expressing cells lose meristematic characteristics, but acquire the potential to delineate boundaries within the IM and to establish adaxial/abaxial polarity for morphogenesis and growth. The resulting network, consisting of 34 significant differentially expressed genes (DEGs) and based on the *Arabidopsis* Transcriptional Regulatory Map (ATRM; [33]), is depicted in Fig. 5 and contains the highest up- and downregulated genes from the categories of meristem maintenance and identity/floral markers, polarity genes and boundary genes listed in Table 1. This network is based on the input data from the founder cell-specific transcriptome dataset and, is therefore, highly relevant.

### Auxin, cytokinin and other hormonal responses

Because interplay between auxin and cytokinin affects cell-type specification in the IM peripheral zone, we analysed the RNA-seq data with respect to cytokinin signalling and auxin biosynthesis, perception, polar transport, and response.

**Fig. 6:**
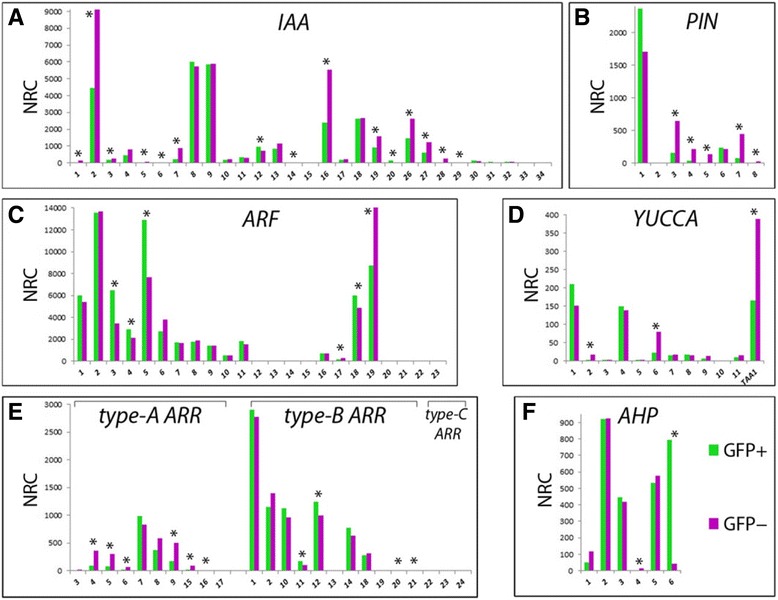
Overview of RNA-seq data for individual transcripts relating to auxin biosynthesis, transport and response, and cytokinin signalling, in terms of normalised read count (NRC) expression levels derived from DESeq2 analysis: **a** *Aux*/*IAA* genes; **b** the *PIN* gene family; **c** the auxin response factor family; **d** genes involved in auxin biosynthesis: the *YUCCA* genes and *TRYPTOPHAN AMINO TRANSFERASE OF ARABIDOPSIS1* (*TAA1*); **e** type-A and type-B cytokinin response regulators; **f** *ARABIDOPSIS HISTIDINE PHOSPHOTRANFERASE* genes. Green bars represent transcript abundance in *DRNL::GFP*-positive protoplasts and pink bars depict the NRC values in *DRNL::GFP*-negative protoplasts. NRC: normalised read counts

For auxin response, we assessed changes in the expression of gene families encoding auxin response factors (ARFs) and their cognate repressor AUX/IAA proteins. Within the *ap1 cal* IM, all 29 AUX/IAA genes were expressed in the *ap1 cal* IM at different levels and some (e.g., IAA15 or IAA33) were very lowly transcribed, below an NRC value of 10 (Fig. 6a). Differences in expression between GFP+/− cells were observed for 15 genes (*p* ≤ 0.01), 14 of which showed a FC ≥ 1.5, including *IAA20* and *IAA29*, which were upregulated, but lowly transcribed, with 130 and 31 NRC, respectively, in GFP+ protoplasts (Fig. 6a). The remaining 13 differentially expressed *AUX*/*IAA* genes were repressed in GFP+ cells and those most affected in transcript numbers were *IAA2*, *IAA16*, *IAA19* or *IAA26*/*PAP1* and *IAA27*/*PAP2*, which were expressed in GFP− protoplasts in a range from 9,097–1,230 NRC and were repressed by about 50 % in GFP+ protoplasts (Fig. 6a).

In contrast, 15 *ARF* genes were expressed: *ARF1–11* and *ARF16–19* and the remaining six ARF family members (*ARF12*–*15* and *ARF20*–*23*; *ARF23* is a pseudogene) were essentially not transcribed. The relative NRCs of all *ARF* genes in GFP+ and GFP− cells are compared in Fig. 6c. The only ARFs that showed significantly altered expression in GFP+ protoplasts that exceeded the FC ≥ 1.5 (*p* ≤ 0.01) threshold were *ARF3/ETT* and *ARF5/MP*, which were up-regulated, and *ARF19*, which was downregulated (Fig. 6c). *ARF5/MP* and *ARF19* encode activating ARFs, whereas *ARF3/ETT* is a truncated repressor ARF. Thus, differences in the AUX/IAA complement in LOFCs exceed those of ARF partners and affect highly and lowly expressed genes.

Among the PIN efflux auxin transporter gene family, *PIN3*, *PIN4*, *PIN5*, *PIN7* and *PIN8* were significantly (FC ≥ 1.5; *p* ≤ 0.01) downregulated in GFP+ protoplasts (Fig. 6b) and among the *YUCCA* (*YUC*) genes and *TRYPTOPHAN AMINO TRANSFERASE OF ARABIDOPSIS1* (*TAA1*), which encode the enzymes of the two-step pathway for auxin biosynthesis, only *YUC2*, *YUC4* and *TAA1* were significantly (FC ≥ 1.5; *p* ≤ 0.01) downregulated (Fig. 6d). Genes encoding the four members of the auxin receptor family (TIR1, AFB2, AFB3 and AFB5), showed virtually no or extremely small statistically non-significant transcriptional differences (FC ≥ 1.5, *p* ≤ 0.01) between GFP+ and GFP− protoplasts (data not shown).

At the level of cytokinin response regulators, genes encoding type-B ARR transcription factors that mediate cytokinin-regulated gene expression were hardly transcriptionally affected i.e., ARR1, ARR2, ARR10, ARR11, ARR12, ARR14 and ARR18 (Fig. 6e). The remaining type-B response regulators, ARR13 and ARR19–21 and type-C ARRs, such as ARR22–24, were not expressed or were very lowly expressed in the *ap1 cal* IM. In contrast, type-A ARRs, which function as negative regulators of cytokinin signalling, were mostly repressed, except ARR7, which was transcriptionally unaffected (Fig. 6e). Therefore, *DRNL*-expressing cells differ from their surrounding neighbours with respect to increased *AHP6* expression and in the downregulation of most negatively acting type-A ARRs.

None of the three cytokinin receptor genes *AHK2*, *AHK3* and *AHK4*/*WOL1*, showed significant transcriptional differences between GFP+/GFP− cells, with NRC values varying between 1,000 and 3,000. Similarly, transcription of the histidine phosphotransfer protein genes *AHP2* (NRC 925/921) *AHP3* (NRC 416/447) or *AHP5* (NRC 575/535) was unaffected and genes encoding AHP1 and AHP4 were only weakly transcribed, although showed slightly reduced expression in GFP+ cells (Fig. 6f). This contrasts with expression of the atypical negative response regulator AHP6 protein, which was highly upregulated (FC = +18.08).

A few significantly DEGs were associated with other hormone pathways (Table 1) and included the upregulation of five *GAox* genes, the upregulation of *BRASSINOSTEROID-INSENSITIVE1-LIKE* (*BRL1*) and the downregulation of *BR ENHANCED EXPRESSION1* (*BEE1*) and *BEE2*.

### Vascular development

There was a striking coordinated down regulation (FC ≥ 1.5; *p* ≤ 0.01) of twelve genes involved in vascular development in GFP+ cells, which are listed in Table 1. These genes included those encoding the peptide EPIDERMALPATTERNING FACTOR LIKE6 (EPFL6) involved in stem elongation, and *CLAVATA3/EMBRYO SURROUNDING REGION* (*CLE*) *41* and *CLE44*, which both encode TRACHEARY ELEMENT DIFFERENTIATION INHIBITORY FACTOR (TDIF). Additional upregulated transcripts were *TDIF-RECEPTOR*/*PHLOEM INTERCALATED WITH XYLEM* (*TDR*/*PXY*), and *WUSCHEL-RELATED HOMEOBOX4* (*WOX4*), which is required for TDIF-induced procambial proliferation. The genes encoding REDUCED IN LATERAL GROWTH1 (RUL1), a receptor-like kinase involved in cambial activity and HIGH CAMBIAL ACTIVITY2 (HCA2) were also affected. Regulators of xylem differentiation that were upregulated in GFP+ protoplasts included *VASCULAR RELATED NAC DOMAIN* (*VND*) *6* and *VND7,* the targets of VND6, *XYLEM CYSTEINE PEPTIDASE* (*XCP*) *1* and *XCP2*, and *NAC SECONDARY WALL THICKENING PROMOTING FACTOR1* (*NST1*)*.*

### Post-transcriptional and epigenetic gene regulation

Because many transcription factors are regulated post-transcriptionally by miRNAs, we analysed the expression of miRNA loci known to be involved in development. Ten different miRNAs were significantly (FC ≥ 1.5; *p* ≤ 0.01) downregulated (Table 1), including *miR164*, known to target *CUC1* and *CUC2*, *miR172* that targets a sub-group of *APETALA2* (*AP2*)-like genes and *miRNA390*, which regulates *TAS3A*, which was also downregulated, and correspondingly, *ETT/ARF3*, the known target gene, was upregulated. Considering epigenetic gene regulation, a group of 10 genes was significantly upregulated that are required for DNA methylation, and *DEMETER-LIKE3* (*DML3*), which can demethylate DNA, was downregulated (Table 1).

## Discussion

The rationale to perform FACS to discriminate *DRNL::* *GFP*-positive cells from *ap1 cal* IMs was that local *DRNL* expression at the flanks of primary or multiple-order IMs exhibits the typical spiral phyllotaxy of LOFCs. Therefore, the characterisation of this cell population by RNA-seq addresses the transcriptional networks operative during the earliest stages of lateral organ initiation and in the *ap1 cal* double mutant background, in a homogeneous reiterative environment of IM identity. RNA-seq analysis revealed more downregulated DEGs than upregulated DEGs in GFP+ protoplasts compared to GFP− protoplasts, considering transcriptional changes of FC ≥ 1.5 (*p* ≤ 0.01). The upregulated DEGs comprised preferential GO categories that supported primordium or floral differentiation, anisotropic growth or cell-cycle progression/division for morphogenesis and that contained several known direct targets of DRNL (*IAA20*, *CUC1*, *AHP6, SHI* [19] and *STYLISH1* (*STY1*) [34]. It remains to be confirmed whether GCC motifs within the promoters of *SHI/STY1* are physical targets of DRNL and contribute to the interpretation of positional information at the IM periphery downstream of *DRNL*. Although the known DRNL target genes have mostly been identified by constitutive *DRNL* overexpression, their demonstrated co-regulation here with *DRNL* activity within LOFCs supports their functional relevance in a defined developmental context with respect to bract or FM identity. Moreover, the RNA-seq data suggest that in addition to *STY1* and *SHI, SHI-RELATED SEQUENCE7* (*SRS7*) and at low transcript levels, *SRS4*, identified as putative *DRNL* targets by [34], might also be functionally relevant in this developmental context.

### The transcriptional network of *DRNL::GFP*-positive cells

The downregulation of the meristematic cell markers *STM* and *BP* in *DRNL*::*GFP+* cells demonstrates loss of meristem identity and is compatible with the acquisition of LOFC fate in a spiral phyllotaxy within the IM peripheral zone. It is accompanied by the transcriptional upregulation of many classical markers such as *AP1*, *CAL*, *LFY*, *UFO* and *AS2*, which are either associated with floral meristem identity or lateral primordia development [9, 35–38]. However, the transcriptional network (Fig. 5) shows that in addition to the transcriptional activation of individual lateral organ markers, entire regulatory gene modules are coordinately upregulated in *DRNL::GFP*+ cells. One well-characterised GRN associated with lateral organ initiation at the IM periphery involves *LFY*, *ANT*, *AIL6* and *FIL* downstream of MP [16, 39], and the subsequent upregulation of *LMI1*, *LMI2*, *TLP8* and *RAX1* by LFY [40–43]. The upregulation of these nine genes in the LOFC transcriptome confirms that this genetic interaction module contributes to the earliest stage of primordia anlagen. Other known interactions within the transcriptional network are the upregulation of *JLO* and *AS2*, whose gene products repress the class I *KNOX* genes *STM* and *BP* [44] and the increase in *AS2*, *YAB5* and *ETT* transcripts, of which the abaxial determinants *YAB5*, and *ARF3* are subsequently adaxially repressed by the *AS1*/*AS2* complex via polycomb-dependent and -independent mechanisms [45].

Two other functional groups of DEGs relate to polarity determination and boundary creation. Lateral organ progenitor cell divisions in the IM occur along a trajectory defined by a centro-peripheral axis [20], which is important for suppressing bract outgrowth and establishing positional information that leads to FM initiation at the adaxial domain of the cryptic bract, and also underlies the unidirectional order of sepal initiation before floral stage 2 [6]. Polarity determinants that are upregulated in LOFCs at the IM periphery include *PUCHI* [4], *BOP1* and *BOP2* [21] and *JAG* [46], which are involved in bract suppression, and several members of the YAB class of transcription factors [47] or *ETT* [24] that affect the ad-/abaxial polarity of lateral organs.

The RNA-seq data furthermore suggest that gene interactions involved in polarity at other developmental stages might function more ubiquitously: for example, *NUBBIN* and *JAG* redundantly promote the polar differentiation of stamen and carpel tissue [48], but their co-upregulation in LOFCs at the IM periphery suggests a potential co-function in these cells. In addition, *RAX1*/*MYB37*, which functions to specify a stem-cell niche for axillary vegetative meristem formation [49], is significantly upregulated and might have an analogous but uncharacterised role in the initiation of FMs as axillary meristems in the cryptic bract axil.

The initial bulging stage in stage 1 flowers, when the bract is morphologically visible [2], involves the creation of a boundary between the bract and the IM. In support of this, a cohort of boundary genes is upregulated in GFP+ cells, including *LBD30*/*JLO* [50], *LBD18* [51], *LOJ* [52] and *CUC1* [53]. The boundary domain possesses its own transcriptional network that represses cell divisions and is characterised by a low level of brassinosteroids [54, 55]; the strong downregulation of the brassinosteroid signalling components BEE1 and BEE2 in *DRNL::GFP*-positive cells indicates that some components of BR signalling are repressed in LOFCs.

### Lateral organ founder-cell specification is associated with subtle changes in auxin or cytokinin biology

Auxin is absolutely required for the FM initiation at the IM and auxin response maxima indicated by the *DR5* reporter are paradigmatically associated with sites of incipient lateral organ initiation [13]. However, auxin-dependent phyllotaxy also depends on a gradient of cytokinin signalling patterned by the negative cytokinin response regulator AHP6 [18], which is a direct target of DRNL and is also co-expressed with *DRNL* in the cryptic bract domain, which is spatially distinct and more distal in the IM to that of auxin response maxima [6]. Therefore, interplay between cytokinin and auxin in two adjacent domains is instructive for the positioning of lateral organs in the IM [56], which presumably relates to FM founder cells in cryptic bract axils. In analogous developmental contexts, such as the patterning of lateral organs from the SAM [57] and axillary bud growth [58], an auxin minimum is required. The RNA-seq data here show limited differences in auxin and cytokinin responses within the LOFC; few *Aux*/*IAA* or *ARR* genes are significantly differentially expressed and many show extremely low transcript levels. Importantly, only three ARFs are differentially expressed: *ETT*, which plays a role in floral polarity [24], and the activator ARFs, *ARF19* and *ARF5*/*MP. MONOPTEROS* is a master regulator, which instigates a gene regulatory network via *LFY* transcription that leads to FM initiation [16, 17, 39,]. The most striking difference in cytokinin signalling and response is the specific upregulation of *AHP6*, which is a pseudohistidine kinase that lacks the characteristic histidine that facilitates phosphorelay during cytokinin signal transduction and is thought to act as a global negative regulator of cytokinin signalling by competing with other AHPs [59]. The transcript levels of type-B *ARR* genes remain unaffected in LOFCs, whereas type-A *ARR* genes, except ARR7, are transcriptionally downregulated.

A similar selective response is seen for auxin efflux carriers, where *PIN3, 4, 5* and *7* transcription is collectively repressed in LOFCs, whereas the abundant *PIN1* or low *PIN6* transcript levels remain essentially unaltered in *DRNL*-expressing cells relative to non-expressing meristematic cells. There is little evidence for global changes in transcription with respect to auxin biosynthesis, transport or perception that accompany LOFC specification at the IM periphery, in striking contrast to the substantial changes in the transcription factor network (Fig. 5) discussed above. Thus, cytokinin or auxin responses for LOFC specification either rely on post-transcriptional control mechanisms or on individual gene activities, such as the large increase in *AHP6*. Alternatively, small transcriptional changes in many AUX/IAA proteins are fundamentally important. Numerically, the downregulation of *ARF19* transcripts is compensated by increased *MP/ARF5* mRNA levels, which might relate to target-gene specificity, although the transcription of the chromatin remodelling factors BRAHMA and SPLAYED, which regulate MP activity [17], remain unaffected in the transcriptome data here.

### Epigenetic and post-translational aspects of the LOFC transcriptome

The downregulation of many miRNAs in LOFCs highlights the relevance of their cell-type-specific spatio-temporal functions in post-transcriptionally regulating the expression of transcription factors during plant development by cleaving mRNAs or blocking translation.

Consistent with the observed upregulation of *CUC1*, miR164, which regulates *CUC1* expression by mRNA cleavage [60] is downregulated. Similarly, the negative regulation of *ETT* transcript accumulation by *miR390* during phase-change [61], which cleaves TAS3A [62], is consistent with a significantly lower *miR390* and TAS3A abundance observed in LOFCs here and the upregulation of *ETT*, suggesting that this regulatory module functions early in LOFCs. Furthermore, *miR172* negatively regulates a sub-set of *APETALA2* (*AP2*)-type genes, including the floral organ identity gene *AP2*, by blocking mRNA translation [63], which is consistent with no significant change in the expression of AP2 or the other *miR172* targets *TARGET OF EARLY ACTIVATION TAGGED* (*EAT*) *1* (*TOE1*), *TOE2*, *TOE3*, *SCHLAFMUTZE* and *SCHNARCHZAPFEN*. Other miRNAs downregulated here potentially regulate *ARF* (*miR160*) [64] and *SPL* genes (*MiR156*) involved in phase change and the regulation of flower-promoting MADS-box genes [65].

The coordinated upregulation of ten genes involved in DNA methylation, particularly the cluster of *VIM* genes [66] and the downregulation of the demethylase, *DML3* [67], suggest that epigenetic gene regulation contributes to the specification of founder cells marked by *DRNL*, in addition to transcriptional changes.

### The RNA-seq data support bract initiation as the initial step of lateral organ development at the IM periphery

Phytomer theory predicates that the earliest event in lateral organ initiation at the IM periphery is bract initiation, followed by the initiation and outgrowth of the FM, which consumes the cryptic bract founder-cell population [68]. The data here contain many upregulated genes associated with floral bract suppression, i.e., *LFY*, *PUCHI*, *BOP1, BOP2*, *UFO* and *FIL*, which allow bract outgrowth when mutated [4, 21, 35, 69, 70], suggesting that bract suppression in wild type is due to the concerted function of several genes that potentially antagonise *JAG* to promote bract development [46]. The interplay between founder-cell recruitment for the bract and FM has been demonstrated genetically using *DRNL* as a marker [6], and functionally, by ablation of the *LFY* expression domain [71]. Expression of *LFY* in the IM encompasses the cryptic bract region and subsequently, the FM, where mobile LFY protein contributes to bract and FM identity [72, 73]. According to the IM/FM mosaic phenotype of *puchi* mutant flowers and the phenomenon of floral reversion, LOFCs at the IM periphery have the potential to newly acquire bract and FM fate or to revert to IM identity. The downregulation of a consortium of genes involved in vasculature differentiation in GFP+ protoplasts suggests that the suppression of vasculature development is an important facet of early lateral organ initiation.

The LOFC transcriptome data here lead to the following conclusions: firstly, the initial LOFC fate acquired at the IM periphery is bract identity, suggested by the upregulation of numerous genes functionally associated with leaf development and ab-/adaxial leaf polarity. This is also supported by the downregulation of the *KNOX* genes *BP* and *STM*, which are antagonised in leaves by *AS1*/*AS2* complex components that are activated in LOFCs, and the initial absence of increased *WUS* and *CLV3* activity, which are reactivated in stage 2 flower primordia [7, 8] to reinstate a stem-cell population in the autonomous FM. Secondly, polar determinants reinforce the autonomy of the LOFC by concomitantly initiating a morphological boundary between the surrounding IM cells. Thirdly, despite evidence for altered interactions within transcription factor networks in LOFCs, the selective changes in auxin signalling observed at the earliest time-point of LOFC initiation are inconsistent with the paradigm that auxin response maxima prepattern sites of lateral organ initiation. However, with respect to cytokinin signalling, the massive co-upregulation of *AHP6* and *DRNL* expression is striking.

## Conclusions

The RNA-seq data obtained following the separation of *DRNL*-expressing LOFCs from the *ap1 cal* IM via FACS provide a unique, robust and cell-type-specific data set that depicts a very early cellular decision towards differentiation in the IM peripheral zone. The differentially expressed transcripts suggest that lateral organ founder-cell specification involves the creation of polarity from the centre to the periphery of the IM and the establishment of a boundary from surrounding cells, consistent with bract initiation. However, contrary to the established paradigm that sites of auxin response maxima pre-pattern lateral organ initiation in the IM, auxin response might play a minor role in the earliest stages of lateral floral initiation. The transcriptome data can not only be used to validate genetic interactions within LOFCs and candidate physical targets of the DRNL AP2-type transcription factor in a cell-type-specific manner, but represent a valuable community resource to address unresolved questions concerning the molecular repertoire that underlies cellular differentiation in the IM peripheral zone, i.e., the specification of bract, sepal or FM founder cells.

## Methods

### Genetic material and growth conditions

To generate material for FACS, we crossed the *ap1 cal* mutant (Nottingham Arabidopsis Stock Centre accession N6161) to the *DRNL::erGFP* marker line and identified transgenic *DRNL::erGFP ap1 cal* progeny in the F_2_ generation. All plants were grown in a controlled greenhouse environment in long-day (16 h light: 8 h dark) conditions.

### Confocal imaging

A Zeiss LSM 700 confocal laser scanning microscope was used to image the *DRNL::GFP* transgenic *ap1 cal* inflorescences and to check the integrity and concentration of *GFP*-expressing protoplasts. GFP was excited at 488 nm and emission was analysed between 490 and 560 nm.

### Fluorescence-activated cell sorting (FACS)

For cell sorting, inflorescence apices of approximately 700 *DRNL::erGFP ap1 cal* plants were harvested four to five weeks after sowing, before the IM showed histological evidence of floral organogenesis. Protoplasts were prepared from inflorescence apices in 30 mL FACS-medium (10 mM KCl, 2 mM MgCl_2_, 2 mM CaCl_2_, 1 g/L BSA, 0.4 g/L MES, 109.3 g/L mannitol, pH 5.5) supplemented with cellulase (20 g/L; Sigma-Aldrich) and pectolyase (1 g/L; Sigma-Aldrich). Free-floating single protoplasts from superficial cell layers were separated from remaining explant tissue by filtration through miracloth (MerckMillipore) and were centrifuged (500 r.p.m., 10 min, 4 °C) and resuspended in 0.5 − 2.0 mL FACS medium to concentrate the suspension to a mean concentration of 7.0 × 10^7^ cells/mL. The GFP-positive (GFP+) and GFP-negative (GFP–) cells were immediately separated on a FACS Vantage SE (Becton Dickinson) sorter for a maximum period of 1 h (36,000 cells s^−1^ flow rate; 100-μm aperture). The sorted protoplasts were directly collected into 9.9 mL DCT lysis solution (Invitrap Spin Plant RNA Mini Kit, Stratec, Berlin) supplemented with 100 μL 1 M DTT and 10 μL RNase Inhibitor (1 U/μL Thermo Fisher Scientific). The protoplast suspension:RNA lysis buffer volume ratio did not exceed 1:5 and frequent mixing during protoplast collection was essential for RNA quality. All other steps followed the manufacturer’s (Stratec) instructions. The number of collected GFP+ protoplasts typically varied between 100,000 to 350,000 per experiment and approximately 500,000 to 700,000 GFP− protoplasts were collected as a negative control. The Invitrap Spin Plant RNA Mini Kit was also used to isolate RNA from *ap1 cal* inflorescence apices.

### Library preparation and deep sequencing

The TruSeq v2 RNA sample preparation kit (Ilumina) was used to prepare cDNA libraries from 200 ng total GFP+ or GFP− RNA. Poly (A)^+^ RNA was purified onto oligo-dT magnetic beads and was fragmented using divalent cations at elevated temperature; RNA fragments were reverse-transcribed using random primers, followed by second-strand cDNA synthesis with RNase H/DNA Polymerase I. After end repair and A-tailing, adapters were ligated and the indexed cDNA products were purified and amplified by PCR (15 cycles) to create the final cDNA libraries. Library quality was validated on a 2200 TapeStation (Agilent Technologies) and individual libraries were quantified on the Qubit System (Invitrogen) prior to pooling and pool quantification via the KAPA Library Quantification kit (Peqlab) and the 7900HT Sequence Detection System (Applied Biosystems). The pooled, indexed libraries were loaded and analysed on an Illumina GAIIx sequencer using the 2 × 100-bp v3 protocol.

### Data analyses

Next-generation sequencing data were analysed using QuickNGS, a high-throughput next-generation sequencing analysis pipeline [74]: Fast QC (Babraham Bioinformatics), as well as read statistics derived from the SAMtools packages, were used to check the quality of the raw data. All software used in QuickNGS version 1.2.0 are summarised at http://athen.cecad.uni-koeln.de/quickngs/web/doc/algorithms.php. Reads were mapped to the *Arabidopsis* reference genome (TAIR v 10; ftp://ftp.arabidopsis.org/home/tair/Genes/TAIR10_genome_release) using TopHat2 [75] using the default parameters and gene quantification was performed using a combination of Cufflinks [76] and the DEseq2 package [77] with genomic annotation from the TAIR10 genome release. Results were uploaded into an in-house MySQL database and merged with annotations obtained with biomaRt from EnsemblGenomes, version 26. The gene lists were filtered according to the fold change (FC) and *p*-value, which were calculated with the DESeq2 package [78] from the Bioconductor project based on library size-normalised read counts (NRC). In contrast, gene expression for the individual samples was calculated by the Cufflinks package and returned as fragments per kilobase of transcript per million mapped read values (FPKM), which represents normalisation by molecule size. To reduce false positives among the differentially expressed genes, we considered only transcripts for which sufficient reads were detected in both the GFP+ and GFP− inputs.

The Principal Component Analysis (PCA) was computed by the R language for statistical computing and was based on log_2_-transformed FPKM values as obtained from the Cufflinks analysis.

The gene ontology grouping of differentially expressed genes was performed at TAIR https://www.arabidopsis.org/tools/bulk/go/index.jsp and the molecular interaction networks were visualised by the Biological Networks Gene Ontology tool (BiNGO) (v. 3.0.3; [32]) in Cytoscape v.3.3 [79]. The appropriate *Arabidopsis thaliana* customised GO annotation file was downloaded from http://geneontology.org (20 June 2015). The BiNGO software calculates the probability of an overrepresentation of genes in a GO-group within the GO hierarchy and includes the false discovery rate (FDR) via the Benjamini and Hochberg correction at a significance level set to a value of 0.05 in our analyses. To construct the transcriptional network within the *DRNL*-marked founder-cell population, we used the Arabidopsis Transcriptional Regulatory Map (ATRM) dataset [33], supplemented with some additional gene–gene interactions from the literature. Transcription factors from the transcriptome dataset that were up- or downregulated more than 1.5-fold at *p* ≤ 0.01 and that were contained within the ATRM dataset were used to construct a network that was visualised in Cytoscape v.3.3.

### Quantitative RT-PCR

For qPCR, RNA (300 to 2,700 ng) was reverse-transcribed using the QuantiTect Reverse Transcription Kit (Qiagen, Hilden, Germany). Real-time PCR experiments were performed using the 7500 Fast Real-Time PCR System by Applied Biosystems. SYBR Select Master Mix (life technologies) and the Fast SYBR Green Master Mix Protocol (Applied Biosystems) were used for the experiments. To verify the RNA-seq data, the expression of 18 genes was assessed by qPCR in up to three biological replicates and three technical replicates; 3– 12 ng cDNA per well was analysed. For evaluation, Ct-values were normalised to those of *ACTIN2* (*At3g18780*) and primer efficiency; primer sequences are listed in Additional file 2. Gene expression levels were calculated using the ddCt method [80]. If the Ct-value could not be determined due to low transcript levels, a value of 40 was assumed for further calculations.

## Additional files

Additional file 1: Comparison of log_2_-transformed expression from qRT-PCR for a subset of 18 up- or downregulated genes from the transcriptome dataset normalised to *ACTIN2* expression and the log_2_ (relative expression) from RNA-seq. (DOC 150 kb)
Additional file 2: Primer sequences and list of genes used for real-time PCR expression analysis. (DOCX 147 kb)

## Abbreviations

DEG(s): Differentially expressed gene(s)
FACS: Fluorescence-activated cell sorting
FM: Floral meristem
GO: Gene ontology
GRN: Gene regulatory network
IM: Inflorescence meristem
LOFC: Lateral organ founder cells
NRC: Normalised read counts
RNA-seq: RNA sequencing
