# Supplementary figures and images for: The founder-cell transcriptome in the Arabidopsis apetala1 cauliflower inflorescence meristem
Source: BMC Genomics. 2016 Nov 3;17:855. doi: 10.1186/s12864-016-3189-x (PMC5093967; doi:10.1186/s12864-016-3189-x)

**Additional file 1.**


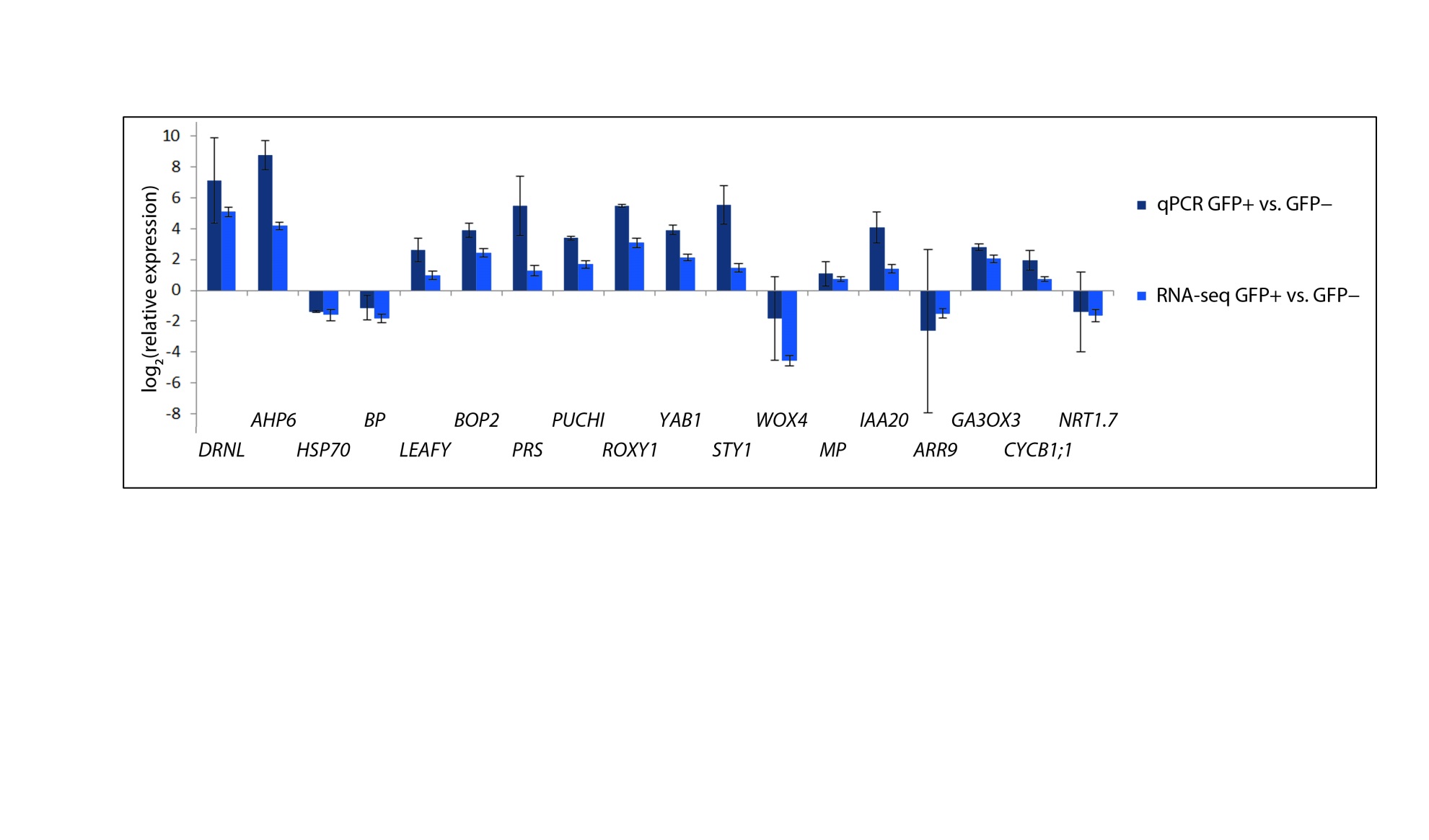

Supplement: Additional file 1: — Comparison of log2-transformed expression from qRT-PCR for a subset of 18 up- or downregulated genes from the transcriptome dataset normalised to ACTIN2 expression and the log2 (relative expression) from RNA-seq. (DOC 150 kb) [file 12864_2016_3189_MOESM1_ESM.doc]
