# Supplementary material for: The founder-cell transcriptome in the Arabidopsis apetala1 cauliflower inflorescence meristem
Source: BMC Genomics. 2016 Nov 3;17:855. doi: 10.1186/s12864-016-3189-x (PMC5093967; doi:10.1186/s12864-016-3189-x)

**Additional file 2.** List of genes and primer sequences used for real-time PCR expression analysis.

|  |  |  | |  |
| --- | --- | --- | --- | --- |
| **Gene ID** | **Alias** | **Primer** |  | **Primer sequence (5´ to 3´)** |
| *At1g24590* | *DRNL* | FP |  | GGTCAAGTACCATTGAGAACGA |
|  |  | RP |  | TTTTGGTAAGAAGCCTTGAACA |
| *At2g46990* | *IAA20* | FP |  | ACGGACCTGAGACTCGGACT |
|  |  | RP |  | CCGCCACATATTCCGCATCC |
| *At5g18560* | *PUCHI* | FP |  | TGCATTGTCCCAAACCACTGC |
|  |  | RP |  | AGGCTTTGCTTGCTACCGGA |
| *At3g51060* | *STY1* | FP |  | CGCGGTGAGAGCGTTCCTAA |
|  |  | RP |  | CACCGCCGATGAACTCACCT |
| *At2g28610* | *PRS* | FP |  | TGGCTTCAACGAGGTGGTGT |
|  |  | RP |  | ACGCCAAGTGAGCTGTGATCT |
| *At1g46480* | *WOX4* | FP |  | AGGACATGTAGGAGCTGGGGAT |
|  |  | RP |  | AGAGGAAAAAGCTCGAGGGTTACA |
| *At5g61850* | *LFY* | FP |  | TTCAAGCACCACCTCCGGTT |
|  |  | RP |  | CTTCGCCGCCGTGTAGAAAC |
| *At1g80100* | *AHP6* | FP |  | f C F CGCAACCTTAGATTATTGTTGAT |
|  |  | RP |  | CCCTACGAGCACCAATGC |
| *At2g41370* | *BOP2* | FP |  | CGTACCCGCTAGCCCAACAA |
|  |  | RP |  | TGTTTCTGCGGCACGATGGA |
| *At3g02000* | *ROXY1* | FP |  | AGAGCGCGGTGGTGATCTTC |
|  |  | RP |  | ATGAGGGCTCGCTGGATGTC |
| *At4g37490* | *CYCB1;1* | FP |  | TGTGCAAAGCTGTTGGCGTA |
|  |  | RP |  | AGGGATCAAAGCCACAGCGA |
| *At1g69870* | *NRT1.7* | FP |  | CTTCCCTTCTACGACCGCGT |
|  |  | RP |  | CACAATGCCCGCAACGATCA |
| *At3g57040* | *ARR9* | FP |  | TCGCAACCGCTGTCTGATCT |
|  |  | RP |  | TCCCCTCTCTGCATTCCCTACT |
| *At4g08150* | *KNAT1* | FP |  | CAACAGCGGTCAACACCGTC |
|  |  | RP |  | TGGGCCTTGTTAGCTCCTCAC |
| *At3g12580* | *HSP70* | FP |  | CAGACAACCAGCCCGGTGTA |
|  |  | RP |  | CGGTACACCTCGTGGAGCAG |
| *At3g18780* | *Actin 2* | FP |  | GTGTTGTTAGCAACTGGGATGA |
|  |  | RP |  | CTCTTCAGGAGCAATACGAAGC |
| *At1g19850* | *MP* | FP |  | AACCGCCAACCGTACTCCTT |
|  |  | RP |  | CTGAGAGCTGAGACCCGCAT |
| *At2g45190* | *YAB1* | FP |  | TCCGATGTGGTTGCTGTACCA |
|  |  | RP |  | ACGGTGCATCTCTCAGCTCC |
| *At4g21690* | *GA3OX3* | FP |  | TCTGGTCCAAACCCGAACCC |
|  |  | RP |  | CACCCCATTCCTCACAGGCT |

**Additional file 2.**


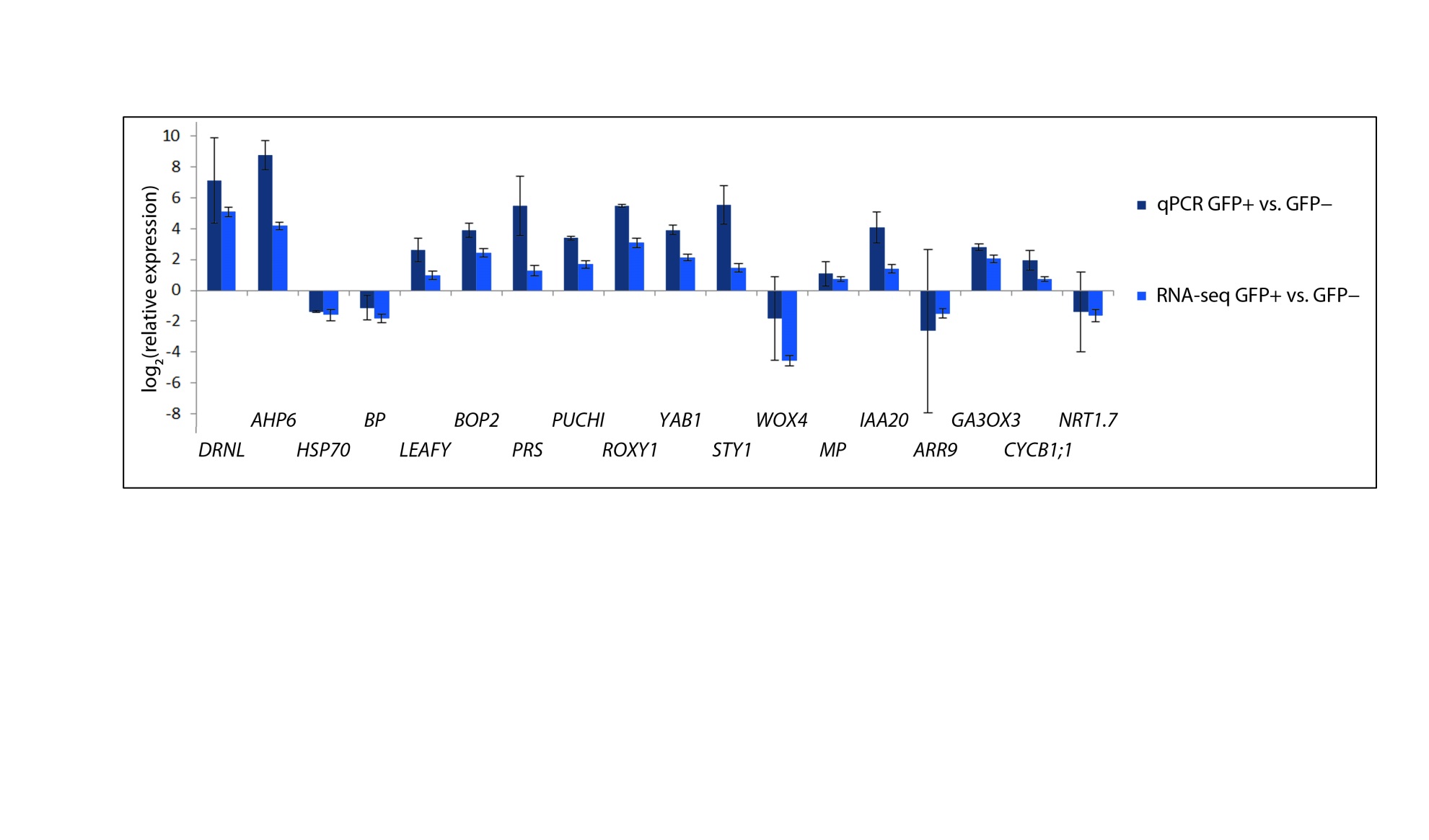

Supplement: Additional file 2: — Primer sequences and list of genes used for real-time PCR expression analysis. (DOCX 147 kb) [file 12864_2016_3189_MOESM2_ESM.docx]
